# Economic impact of robotic liver resection: systematic review of costs, resource utilization, and hospital stay

**DOI:** 10.1007/s00464-026-13132-6

**Published:** 2026-07-29

**Authors:** Mohammed Ibrahim, Mikolaj R. Kowal, André L. Mihaljevic, Perparim Limani, Peter Lodge, Philipp Kron

**Affiliations:** 1 University Clinic for General, Visceral and Transplantation Surgery, University Hospital Tübingen, Tübingen, Germany; 2 Leeds Institute of Medical Research, Faculty of Medicine and Health, University of Leeds, Leeds, England; 3 Department of General and Visceral Surgery, Kantonsspital Aarau, Aarau, Switzerland

**Keywords:** Liver disease, Robotic surgery, Robotic liver resection, Laparoscopic liver resection, Open liver resection, Cost-effectiveness

## Abstract

**Background:**

The robotic approach is being increasingly utilized for liver resections. Patient benefits from robotic liver resection (RLR) have been observed; however, their cost–benefit is yet to be confirmed. This systematic review aimed to determine the costs of RLR compared to laparoscopic (LLR) or open liver resections (OLR).

**Method:**

A systematic search of MEDLINE, EMBASE, and Cochrane databases was performed in October 2025. Non-randomized studies of adult patients undergoing RLR were included. The narrative data synthesis focused on total, intraoperative and indirect costs, and length of stay (LOS). Where study heterogeneity was deemed acceptable (I^2^ < 75%), meta-analyses were performed. Quality assessment was performed using ROBINS-I and the Drummond economic evaluation checklist.

**Results:**

32 studies were included, involving a total of 16,592 patients. In RLR vs. OLR comparisons, most studies (*n* = 13/18, 72.2%) reported equivalent or lower total costs for RLR. In RLR vs. LLR comparisons, most studies (*n* = 10/18, 55.6%) reported higher total costs for RLR, confirmed by meta-analysis (+ 5,250 PPP-adjusted USD, 95% CI + 3,360 to + 7,150). A minority of studies (*n* = 8/32, 25%) addressed indirect costs or costs adjusted for case volume or complexity. When assessed, cost differences between RLR and LLR decreased in high-volume centers. No study performed a formal cost-effectiveness analysis.

**Conclusion:**

Available evidence suggests laparoscopy remains the least costly approach. The cost of RLR may decrease in high-volume centers, complex resections, and with increasing diversity of robotic platforms. Standardization of cost reporting and quality of health economic analyses must be improved to provide definitive cost-effectiveness evaluations.

**Graphical abstract:**

**Figure:**
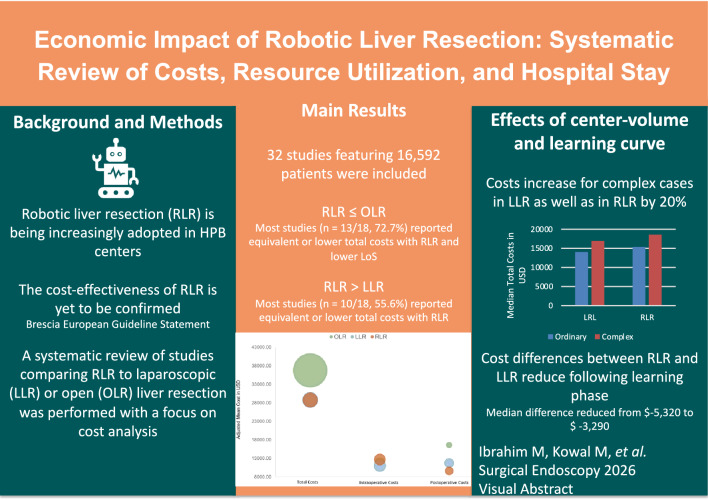

Liver resections are a major management strategy for benign and malignant liver diseases. In the context of primary and secondary liver malignancies, liver resections can provide a curative option for patients. In the example of the most common tumor type, colorectal liver metastasis (CRLM), liver resection combined with systemic anti-cancer therapy can yield 5-year survival rates of up to 50% for patients [1–3]. Technological advances in surgery have led to the development of the robotic approach, facilitating novel minimally invasive techniques. Accordingly, the robotic approach has been adopted for liver resections, showing improved patient outcomes such as reduced LOS, blood loss, and postoperative pain [4–6]. With global cancer rates on the rise, predicted to reach 35 million new cases per year by 2050, it is imperative to develop surgical techniques which yield the best possible patient outcomes [7].

The adoption of robotic liver resection (RLR) is advocated by professional groups, such as the Brescia European guidelines on minimally invasive liver surgery [8]. The use of robotic surgery for left and right hepatectomy and minor liver resections is supported by clinical evidence. However, the guidelines were unable to comment on the current cost-effectiveness of RLR due to the paucity of available data. With the economic pressure on healthcare providers and the aim of a widespread adoption of RLR, cost must be considered against the clinical advantages. This systematic review aimed to evaluate the current cost implications of RLR in adult patients, compared to LLR or OLR.

## Methods

### Study design and definitions

The study protocol was developed in accordance with the PRISMA and AMSTAR 2 guidelines and was prospectively registered with PROSPERO (registration number CRD420251162341) [9, 10].

### Selection criteria

All manuscripts meeting study-type criteria which featured a cost analysis for the use of RLR as an intervention for adult patients undergoing surgery for benign or malignant liver disease were included. Study-type criteria included cohort studies and randomized control trials (RCTs). Systematic reviews were used to identify further relevant primary research studies but were not included in the analysis directly. We excluded non-English publications, case reports/series, conference abstracts/editorials/letters/reviews, non-human studies, duplicate/overlapping cohorts, and studies without extractable data for the predefined outcomes.

### Systematic literature search

Embase (Ovid), MEDLINE (Ovid), and Cochrane Library databases were systematically searched. All studies published until September 2025 were considered for inclusion in this systematic review. All identified studies were reviewed against the inclusion and exclusion criteria to assess eligibility. Referenced studies within identified literature were accessed and also considered for inclusion. Screening was performed by two independent investigators (MK and MI) and studies identified were analyzed for relevance to the systematic review prior to full inspection. Any discrepancies between the independent investigators were addressed by a third senior investigator (PK) until consensus was achieved. The search strategies used are displayed in full in Table 6.

### Primary and secondary outcomes

Primary outcomes involved costs associated with the surgical procedure and inpatient admission related to the primary surgery and specifically included the total cost of procedure, total cost of inpatient admission, and length of stay (LOS). The secondary outcomes focused on the costs attributed indirectly from the use of robotic surgery to explore the current economic factors in RLR.

### Data extraction

Two independent investigators (MK & MI) extracted data in duplicate using a standardized data collection form. The senior author (PK) was consulted on any discrepancies. Data were extracted on study characteristics (number of patients, study type, country of origin), patient characteristics (pathology involved), intervention (minor or major liver resection, center volume, type of robotic platform used), and primary and secondary outcomes. When available, additional data such as postoperative costs or intraoperative costs were collected.

### Data synthesis

To summarize the current cost analyses for RLR, mean costs per procedure and absolute median delta differences for comparison groups were reported. An exploratory sub-analysis was conducted to account for learning-curve and center-volume effects by restricting the analysis to studies from self-reported high-volume centers that reported exclusion of early cases due to the learning curve. All cost estimates were adjusted to January 2026 values. Country-specific CPI data from official national statistical agencies were used to inflation-adjust costs from the original study year to January 2026. Subsequently, costs were converted to U.S. dollars using market exchange rates or to PPP-adjusted international dollars using country-specific implied PPP conversion rates from the IMF World Economic Outlook database; OECD guidance was used as the methodological reference for PPP. Costs already reported in U.S. dollars were adjusted using the U.S. CPI. When applicable, treatment effects were displayed in odds ratios (ORs) or risk ratios (RRs) with 95% confidence intervals (CIs) for dichotomous data. Continuous data treatment effects were expressed as mean differences with 95% CIs. Statistical significance was evaluated using the Mann–Whitney U test for two-group comparisons. Alongside the traditional approach to reporting data, a structured narrative synthesis was conducted in line with the Guidance on the Conduct of Narrative Synthesis in Systematic Reviews from the Economic and Social Research Council [11].

Due to anticipated study heterogeneity, a formal meta-analysis of all effect estimates was not planned. Exploratory meta-analyses were performed for LOS, intraoperative costs, and total costs. Heterogeneity across studies was assessed using the I^2^ statistic. An I^2^ value below 75% was pre-specified as indicating acceptable heterogeneity for pooling in the primary analyses and was reported in the results. Where I^2^ exceeded this threshold, pooled estimates were still calculated using a random-effects model but are explicitly labeled as exploratory, given the substantial between-study variance, and should be interpreted with caution. Continuous outcomes were pooled as mean differences (MDs) with corresponding 95% confidence intervals (CIs), comparing RLR resection with either OLR or LLR. When studies reported medians with interquartile ranges or ranges, means and standard deviations were estimated using established methods described by Hozo et al. and Wan et al. [12, 13]. Missing standard deviations were imputed in sensitivity analyses using the median coefficient of variation derived from studies with available variance data. All statistical analyses were performed using R statistical software (Version 4.6 for Mac, R Foundation for Statistical Computing, Vienna, Austria). Meta-analyses were conducted using the meta package.

### Quality assessment

Quality assessment was conducted independently by MK and MI. Any disagreements were consulted with the senior author PK. The ROBINS-I tool was used for non-randomized studies [14]. The risk of bias was assessed as “low risk,” “moderate risk” or “serious risk,” for each study. The results were depicted graphically using a visualization tool by McGuinness et al. [15]. Given the focus of this study on cost-analysis outcomes, the Drummond checklist was also used for quality assessment, a tool designed for the critique of economic evaluations [16].

## Results

### Included and excluded studies

**Fig. 1:**
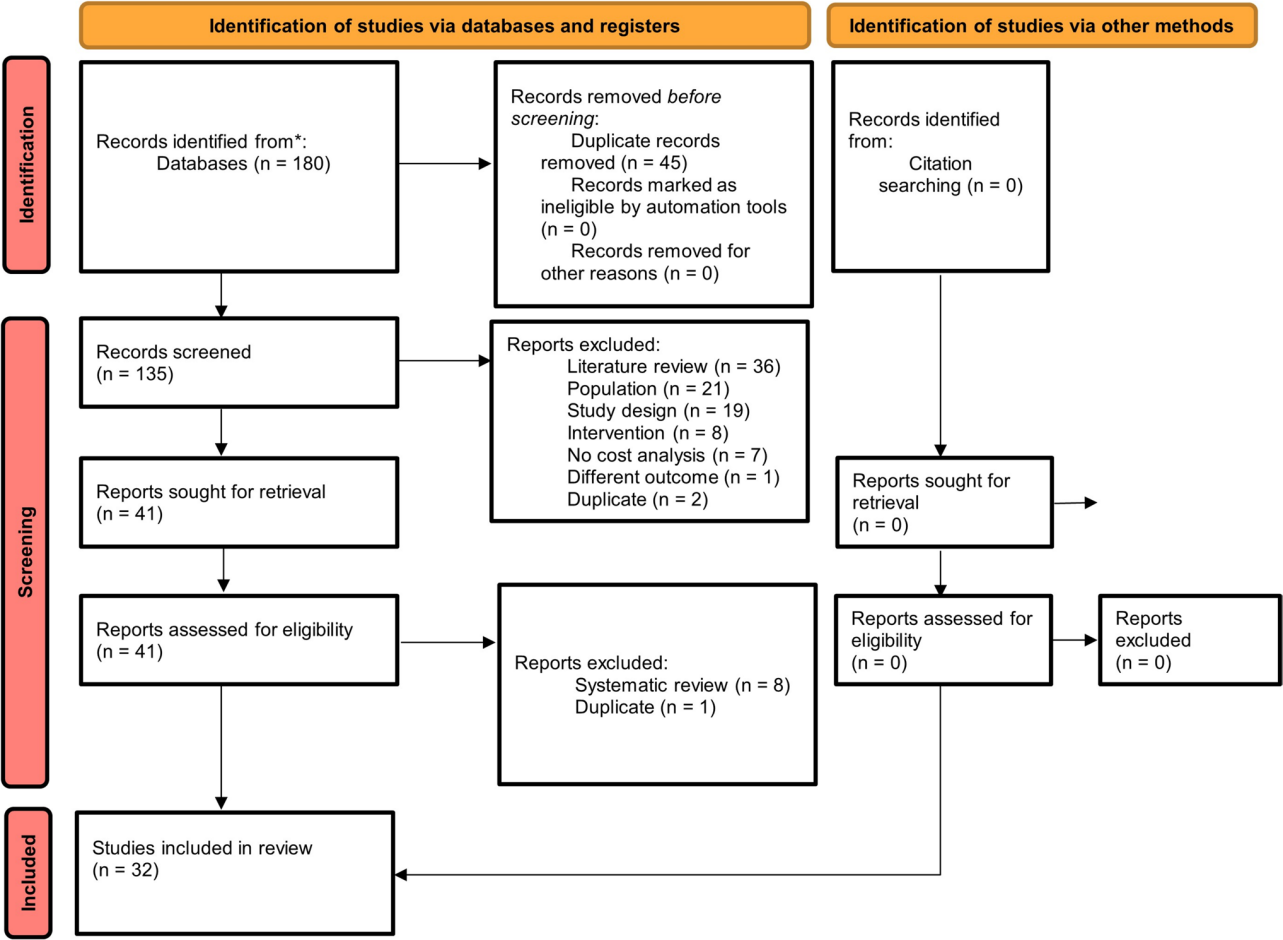
PRISMA flow diagram of study selection

The search strategy returned 135 articles after removing duplicate studies. Following abstract screening, 94 articles were excluded. Full-text reviews excluded 9 studies. The 32 included studies subsequently underwent data extraction. The selection process together with reasons for exclusion is outlined in the PRISMA flowchart in Fig. 1.

### Study characteristics

All 32 included studies were non-randomized (Table 1). 3 studies included data from national registries (*n* = 3/32, 9.4%). They were internationally distributed predominantly from the USA (*n* = 13/32, 40.6%), Europe (*n* = 8/32, 25%), and China (*n* = 7/32, 21.9%), and conducted between 2011 and 2025, with the majority within the last 5 years (*n* = 20/32, 62.5%). 11 Studies compared OLR with RLR (34.3%), 13 LLR and RLR (40.6%), and 8 (25%) compared all three techniques. In total 16,592 patients were included in the entirety of the studies. The Intuitive DaVinci System was the only reported robotic platform in the studies.

**Table 1:** Study and patient characteristics

References	Year	Origin	Study type	n			Patient characteristics				Outcomes assessed
				O	L	R	Benign n/%	Malignant n/%	Minor hepatectomy n/%	Major hepatectomy n/%	
Huang et al.	2025	Taiwan	RS	373	111	78	0	562	484	64	b, h
Roualt et al.	2025	France	RS	18	10	43	NA	NA	NA	NA	a, c, h, e, g, i
Hawksworth et al.	2025	USA	RS	100	NA	100	63	137	20	80	a, b, c, d,e, i
Pilz da Cunha et al.	2024	Netherlands	RS	NA	155	143	76	222	279	19	a, b, c, e,g, h, i
Azimuddin et al.	2024	USA	RS	61	NA	61	3	119	122	0	a, b, c, d, e, f
Elshaer et al.	2024	UK	RS	251	21	60	NA	NA	NA	NA	c, h
Rayman et al.	2023	USA	PS	49	NA	49	NA	NA	40	58	a, b, c, e
Chen et al.	2023	China	RS	NA	41	41	36	46	82	NA	a, c, h
Knitter et al.	2023	Germany	RS	62	59	25	26	120	0	146	a, b, c, e
Winckelmans et al.	2023	Belgium	RS	NA	452	177	72	557	570	59	a, b, c, h, i
Xie et al.	2023	China	RS	83	NA	88	NA	NA	NA	NA	a, c
D’Hondt	2022	Belgium	RS	NA	120	71	67	215	282	29	a, b, c, e,h, i
Aziz et al.	2022	USA	RS	202	202	101	NA	NA	NA	NA	a, b, c, d
Miller et al.	2022	USA	RS	NA	227	227	NA	NA	NA	NA	a, b, c, e
Cai et al.	2022	China	RS	NA	27	25	25	27	NA	NA	a, c
Shapera et al.	2022	USA	RS	104	NA	267	NA	NA	NA	NA	a, c, e
Zhu et al.	2022	China	RS	NA	176	82	117	141	NA	NA	a, b, c
Hawksworth et al.	2021	USA	RS	32	NA	26	20	38	31	27	a, b, c, d, i
Stewart et al.	2021	USA	RS	41	NA	46	10	77	87	0	a, b, c, e
Packiam et al.	2021	USA	RS	NA	18	11	25	14	NA	NA	c, e, g
Mejia et al.	2019	USA	RS	NA	85	35	95	119	160	54	a, b, c, e
Hu et al.	2019	China	RS	NA	54	58	31	81	112	0	a, c
Cortolillo et al.	2019	USA	RS	10,146	520	204	3,826	7044	NA	NA	a, c, d, i
Wu et al.	2019	Taiwan	RS	50	NA	50	24	76	49	51	a, b, c, e
Shu et al.	2018	China	RS	52	NA	26	78	0	NA	NA	a, c
Salloum et al.	2017	France	RS	NA	80	16	39	57	96	0	a, b, c, e
Daskalaki et al.	2017	USA	RS	54	NA	67	NA	NA	70	53	a, c, d, e
Croner et al.	2016	Germany	RS	53	19	10	18	64	82	0	a, c
Kim et al.	2016	Korea	RS	NA	31	12	12	31	43	0	a, c
Sham et al.	2016	USA	RS	88	NA	71	15	144	116	43	a, b, c, e
Yu et al.	2014	Korea	RS	NA	17	13	15	15	NA	NA	a, c
Ji et al.	2011	China	RS	32	20	13	NA	NA	56	9	a, c

Minor hepatectomy: Liver resection involving less than 4 segments; Major hepatectomy: Liver resection involving 4 or more segments; *NA* not available, *PS* prospective study, *RS* retrospective study; *O* open; *L* laparoscopic, *R* robotic

Outcomes legend: a: total costs; b: intraoperative costs; c: length of stay; d: readmission costs; e: postoperative care costs; f: outpatient Costs; g: indirect cost; h: consumable cost; i: initial hospitalization costs

### Total costs

**Fig. 2:**
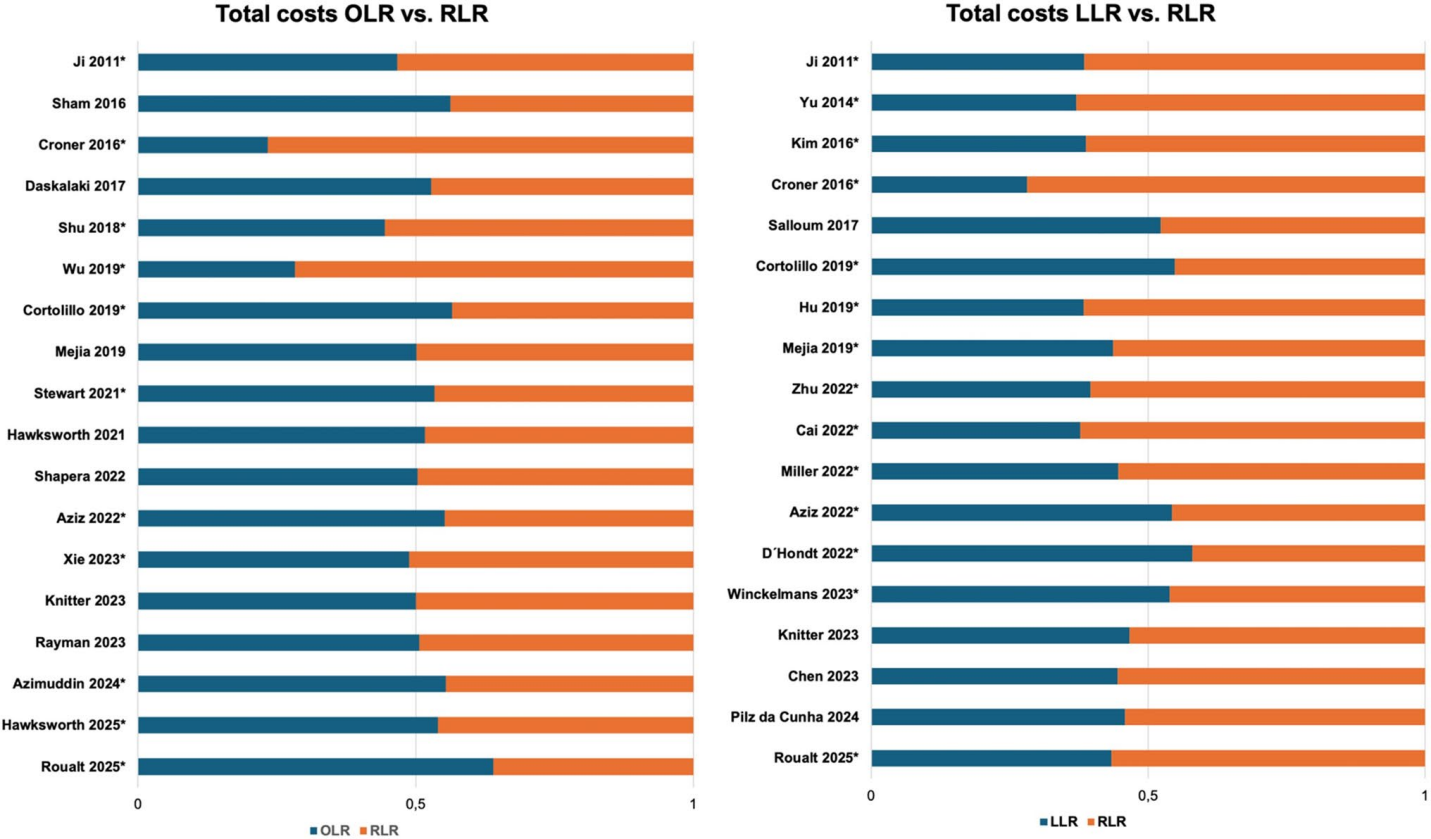
Cost ratios of total costs

29 of 32 studies (90.6%) reported on total costs, including the costs of primary hospitalization, procedure, and/or readmissions. Of these 29 studies, 18 compared OLR with RLR, and 18 compared LLR with RLR. 7 studies assessed all three techniques and were therefore included in both comparisons. In the OLR vs. RLR comparison, 7 studies (*n* = 7/18, 38.9%) reported significantly lower total costs for RLR [17–23], 5 (*n* = 5/18, 27.8%) reported lower costs for OLR [24–28], and 6 (*n* = 6/18, 33.3%) found no statistically significant difference [29–34]. In contrast, in the LLR vs. RLR comparison, 10 studies (*n* = 10/18, 55.6%) reported LLR as significantly less costly than RLR [19, 27, 28, 34–40], 4 (*n* = 4/18, 22.2%) reported lower costs for RLR [21, 23, 41, 42], and 4 (*n* = 4/18, 22.2%) found no difference [30, 43–45]. Across studies, higher costs for RLR were most commonly attributed to robot-specific intraoperative cost components (e.g., disposable instruments, maintenance, and longer operative times). Potential offsetting costs, particularly versus OLR, were described through shorter LOS and reduced postoperative resource use (e.g., pharmacy and laboratory costs), although these drivers were not uniformly captured across cost definitions. Overall, earlier studies (predominantly pre-2021) more often favored OLR in terms of total costs, whereas more recent studies more frequently reported cost neutrality or lower costs for RLR versus OLR. Compared with LLR, postoperative outcomes and downstream resource use were often similar, and differences in total cost were largely driven by higher intraoperative robotic costs. More recent studies reported cost neutrality between RLR and LLR. The distribution of studies over time and corresponding cost ratios for OLR vs. RLR and LLR vs. RLR are shown in Fig. 2.

**Fig. 3:**
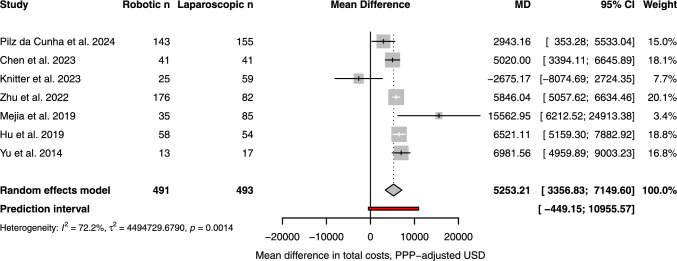
Forest plot comparing total costs between RLR and LLR

The exploratory meta-analyses revealed high levels of heterogeneity (I^2^ > 75%) when analyzing total costs for RLR vs. OLR and were therefore not reported. Acceptable levels of heterogeneity were observed for RLR vs. LLR analyses and were reported. RLR was associated with significantly higher total costs compared with LLR in the primary analysis of seven studies (MD + 5,250 PPP-adjusted USD, 95% CI + 3,360 to + 7,150; *p* < 0.0001; I^2^ = 72.2%) (Fig. 3). Although below our pre-specified threshold, heterogeneity remained substantial (I^2^ = 72%), and pooled cost estimates should be considered hypothesis-generating rather than definitive.

### Intraoperative costs drivers

17 of 32 studies (53.1%) reported on intraoperative costs. 9 of these studies (*n* = 9/17, 52.9%) reported on OLR vs. RLR and 10 (*n* = 10/17, 58.8%) reported on LLR vs. RLR. 7 studies (*n* = 7/9, 77.8%) showed reduced intraoperative costs in RLR compared to OLR. [18, 20, 22, 25, 30, 31, 46]. Only Aziz et al., who performed a retrospective analysis of the 2015 National Readmission Database (NRD), showed significantly lower costs index surgery costs for the robotic approach in comparison with open and laparoscopic surgery (OLR $52,490 LLR $50,330, RLR $45,590, *p* < 0.01)[23]. Stewart et al. found no difference between open and robotic surgery [17].

6 studies (*n* = 6/10, 60%) reported higher intraoperative costs in RLR compared to LLR [30, 35, 37, 41, 45, 46]. 2 studies (*n* = 2/10, 20%) showed an intraoperative cost benefit of RLR [23, 42]. Higher blood loss, operative time, and use of costly materials were identified as main cost drivers across all studies. The robotic approach was often associated with lower blood loss compared to open surgery. However, longer operations and more expensive materials were major cost drivers in the robotic groups.

The exploratory meta-analyses revealed high levels of heterogeneity (I^2^ > 75%) when analyzing all intraoperative costs and were therefore not reported.

### Postoperative cost drivers

**Fig. 4:**
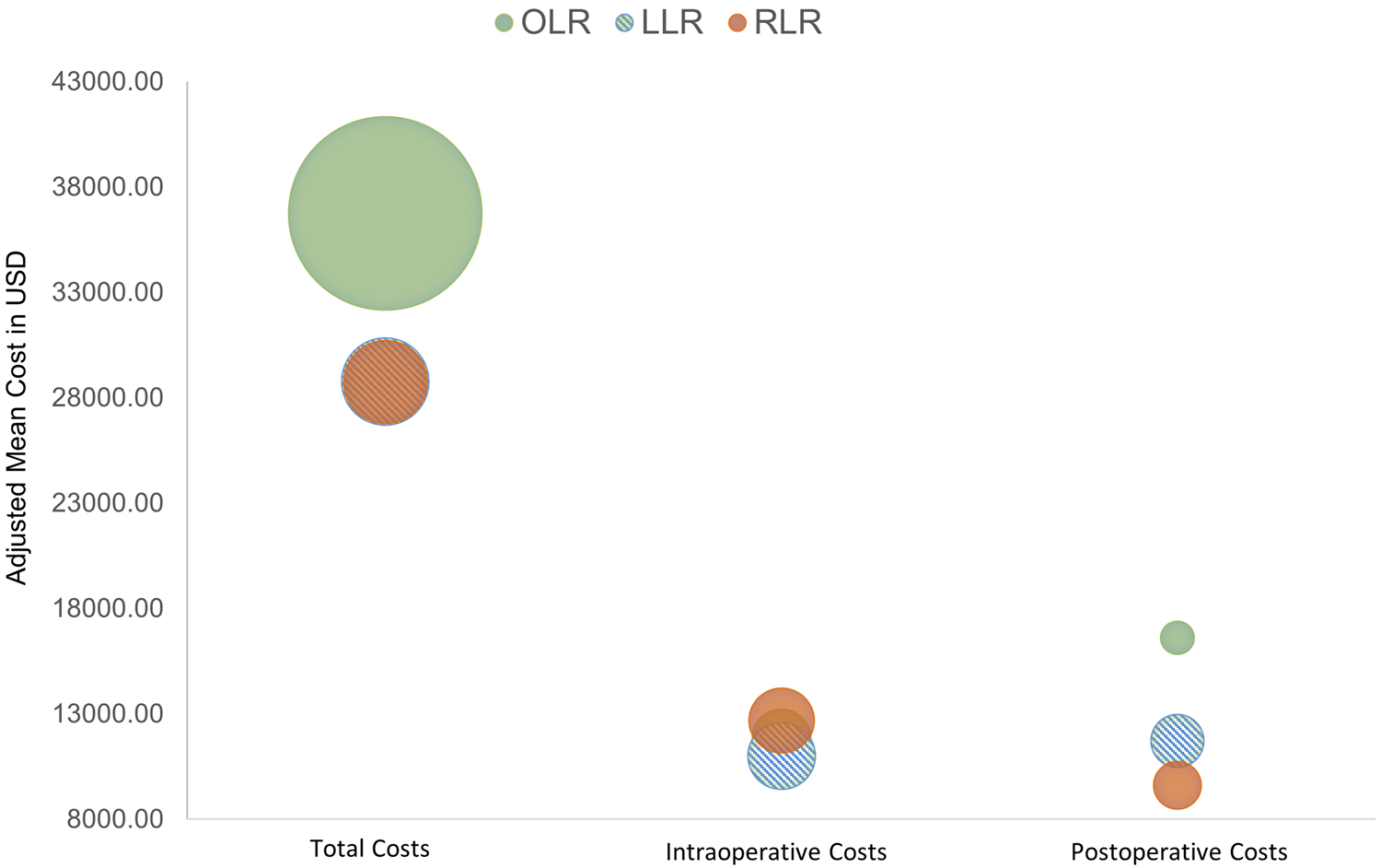
Average total, intraoperative and postoperative costs per surgical approach

Length of stay (LOS) was reported in 27 of 32 included studies (84.4%) and was the major cost driver postoperatively (Table 2). Among studies comparing RLR with OLR (*n* = 14), 10 studies (71.4%) reported a significantly shorter LOS after RLR [17, 18, 20–22, 24, 30, 31, 33, 47]. Four early studies (all published before 2018) reported no significant difference between OLR and RLR [26–28, 32]. Studies that modeled costs, identified LOS and complication burden as major cost drivers, helping explain why the robotic approach can offset higher intraoperative expenses, when compared to open surgery [20, 30]. In contrast, comparisons between RLR and LLR (*n* = 16) showed lower and less consistent differences in LOS. Most studies (*n* = 10/16, 62.5%) reported no significant difference. However, several recent studies with subgroup analyses favored RLR. Winckelmanns et al. reported a shorter median postoperative LOS after RLR (3 days, IQR 1–3) compared with LLR (4 days, IQR 3–6; *p* < 0.001) [41]. The average total, intraoperative and postoperative costs per surgical approach are represented graphically in Fig. 4.

**Table 2:** Average length of stay and average secondary cost outcomes (converted to United States Dollar)

References	Length of stay (days)			Indirect costs		
	O	L	R	O	L	R
Huang et al.	N/A	N/A	N/A	N/A	N/A	N/A
Roualt et al.	N/A	N/A	N/A	N/A	N/A	985/case
Hawksworth et al.	7.4	N/A	2.9	N/A	N/A	N/A
Pilz da Cunha et al.	N/A	3	3	N/A	N/A	2132/case
Azimuddin et al.	3	N/A	2	N/A	N/A	N/A
Elshaer et al.	N/A	5	3	N/A	N/A	N/A
Rayman et al.	6	N/A	4	8753/case	N/A	7899/case
Chen et al.	N/A	7.4	5.6	0	0	5735/case
Knitter et al.	14	9	9	N/A	N/A	N/A
Winckelmans et al.	N/A	4	3	N/A	N/A	N/A
Xie et al.	9	N/A	7	N/A	N/A	N/A
D’Hondt	N/A	3/5**	2	N/A	N/A	N/A
Aziz et al.	N/A	N/A	N/A	N/A	N/A	N/A
Miller et al.	N/A	3	4	N/A	N/A	N/A
Cai et al.	N/A	8	8	N/A	N/A	N/A
Shapera et al.	6	N/A	4	8129/case	N/A	7723/case
Zhu et al.	N/A	4	4	N/A	N/A	N/A
Hawksworth et al.	5	N/A	2.5	N/A	N/A	N/A
Stewart et al.	6.2	N/A	2.2	N/A	N/A	N/A
Packiam et al.	N/A	3	4	N/A	4,408/case	6553/case
Mejia et al	N/A	N/A	N/A	N/A	N/A	150,000/year
Hu et al.	N/A	4.4	4.3	N/A	N/A	N/A
Cortolillo et al.	7.6	6.8	4.5	N/A	N/A	N/A
Wu et al.	N/A	N/A	N/A	N/A	N/A	N/A
Shu et al.	17.81	N/A	13.54	0	0	5000/case
Salloum et al.	N/A	7	6	N/A	N/A	N/A
Daskalaki et al.	9.2	N/A	6	N/A	N/A	N/A
Croner et al.	10	8	7	N/A	N/A	N/A
Kim et al.	N/A	7	7	N/A	N/A	N/A
Sham et al.	6.5	N/A	4.2	N/A	N/A	N/A
Yu et al.	N/A	9.5	7.8	N/A	N/A	N/A
Ji et al.	9.6	5.2	6.7	N/A	N/A	N/A

*LOS* length of stay; *N/A* not available, *O* open, *L* laparoscopic, *R* robotic

^*^Two laparoscopic groups (initial/mastery group)

**Fig. 5:**
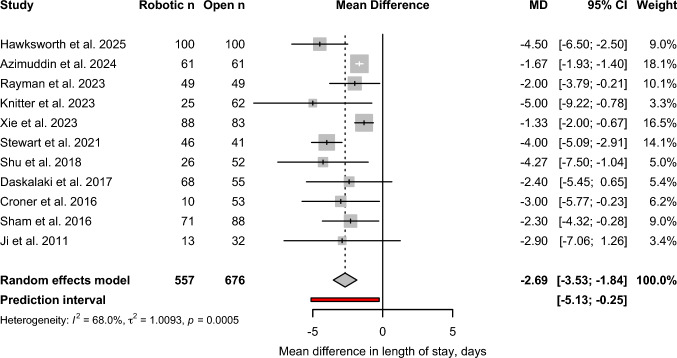
Forest plot comparing length of stay between RLR and OLR

The exploratory meta-analyses revealed high levels of heterogeneity (I^2^ > 75%) when analyzing LOS for RLR vs. LLR and were therefore not reported. Acceptable levels of heterogeneity were observed for RLR vs. OLR analyses and were reported. In the primary random-effects meta-analysis of 11 studies, patients undergoing RLR experienced shorter LOS than patients undergoing OLR. (MD − 2.69 days, 95% CI − 3.53 to − 1.84; *p* < 0.0001; I^2^ = 68.0%) (Fig. 5). This effect remained statistically significant across sensitivity analyses and leave-one-out analysis.

### Indirect costs

Indirect costs were addressed in 8 studies (*n* = 8/32, 25%) [19, 26, 33, 34, 43, 44, 47, 48]. Indirect costs were analyzed due to their relevance to robotic platforms, as they include high, fixed costs that are not captured in routine perioperative accounting, such as maintenance costs or the acquisition of the robotic platform. Additional costs ranged between $1,300 and $12,000 per procedure, with an average extra cost of $3,980 per procedure. Packiam et al. included an indirect cost calculation in their retrospective single-center comparison of robotic vs. pure laparoscopic left lateral sectionectomy (LLS), including 11 robotic and 18 laparoscopic cases. Their indirect cost calculation was based on the dual-console da Vinci model used at their institution: purchase cost $2.2 million ($3,3 M CPI adjusted), annual maintenance $150,000 ($227,410 CPI adjusted), 10-year lifespan with an estimated use for 260 robotic cases/year, resulting in an estimated $2,157.36 indirect cost per robotic case [48]. While direct OR supply costs were similar between the two groups ($7,600 vs. $6,540 *p* = 0.401), the addition of purchase/maintenance allocation made RLR significantly more expensive ($9,720 vs. $6,540 *p* = 0.021). Pilz da Cunha et al. showed similar results, with RLR becoming significantly more expensive once purchase and maintenance were added, increasing total cost by roughly $2,940 (~ €1,840) per case [43]. Rouault et al. explicitly modeled a fixed robot cost by amortizing purchase and maintenance over time and dividing by annual robot volume, estimating a per-case platform cost of approximately $1,300 (~ €850) per operation. Their interpretation was that in a well-established, high-volume center, reductions in LOS and complications can outweigh the robot’s fixed-cost allocation [19]. It is important to note that the analysis still depends on the volume and costing assumptions.

### Case complexity

Six studies (*n* = 6/32, 18,8%) evaluated costs in complex liver resections, defined as major hepatectomies, posterosuperior segment resections, or tumors in close proximity to major vessels [30, 34, 35, 39, 43, 44]. All six comparative studies assessed laparoscopic versus robotic approaches in complex cases. Across most studies (*n* = 4/6, 66,7%), increasing procedural complexity was not associated with a significant cost difference between the laparoscopic and robotic approaches. Costs increased both for LLR and RLR from ordinary to complex cases by approximately 20%. The effects of case severity on RLR costs vs. LLR costs are shown in Table 3.

**Table 3:** The effects of surgical approach, case complexity, and center volume on total, intraoperative, and readmission costs

RLR costs comparison to	Studies favoring RLR	Studies favoring LLR/OLR	Median cost delta	Main costs driver
Total costs OLR	7/18, 38.9%	5/18, 27.8%	+ 900 $	LOS, complication rates, readmission rates
Total costs LLR	4/18, 22.2%	10/18, 55.6%	− 5320 $	Operative time, indirect robotic costs
Total costs "complex cases" LLR	0/6, 0%	2/6, 33.3%	− 5010 $	Case complexity
Intraoperative costs OLR	1/9, 11.1%	7/9, 77.8%	− 3560 $	Operative time, indirect robotic costs
Intraoperative costs LLR	2/10, 20%	6/10, 60%	− 1070 $	Operative time, indirect robotic costs
Initial admission OLR	6/6, 100%	0/6, 0%	+ 5900 $	ICU and ward costs, blood transfusion
Initial admission LLR	2/6, 33.3%	1/6, 16,7%	+ 260 $	NA
Readmission OLR	1/6, 16,7%	0/6, 0%	NA	Complication rates, medication costs, LOS
Readmission LLR	1/3, 33,3%	0/3, 0%	NA	NA
Total costs learning curve + high volume OLR	3/8, 37,5%	0/8, 0%	+ 1660 $	Conversion rates, complication rates
Total costs learning curve + high volume LLR	1/6, 16,7%	2/6, 33,3%	− 3290 $	Operative time, indirect robotic costs
Intraoperative costs learning curve + high volume OLR	1/6, 16,7%	3/6, 50%	− 3560 $	Operative time
Intraoperative costs learning curve + high volume LLR	1/4, 25%	2/4, 50%	− 1310 $	Operative time

*LOS* length of stay, *N/A* not available, *OLR* open liver resection, *LLR* laparoscopic liver resection, *RLR* robotic liver resection

Five studies (*n* = 5/32, 15.5%) included learning-curve effects on costs. The definition of the learning phase across the five studies ranged from 15 to 50 initial cases. These were removed from the analyses to explore volume effects following the learning phase [19, 20, 35, 43, 44]. 9 studies (*n* = 9/32, 28.1%) mentioned conducting RLR in a high-volume center [19, 20, 30–33, 35, 42–44, 47].

In the limited subset of studies reporting data related to learning curve or center volume, the median difference in total costs between RLR and OLR shifted toward lower costs for RLR with a median delta of $1,660. However, this finding should be interpreted as exploratory, as only five studies provided relevant data, definitions of learning curve and center volume varied substantially, and quantitative pooling was not feasible. Compared with LLR, cost differences associated with RLR appeared to attenuate in studies accounting for learning curve effects or high-volume center experience, but the small number of studies and methodological heterogeneity preclude firm conclusions.

Roualt et al. compared open, laparoscopic, and robotic liver resection between 2018 and 2021 and found that robotic costs declined over time, becoming comparable to open and laparoscopic surgery due to reductions in indirect costs, complications, and hospital stay. They also suggested that robotic surgery allowed complex cases that would otherwise require an open approach to be managed minimally invasively, thereby reducing costs. D’Hondt et al. examined costs during the initial mastery phase of robotic liver surgery and after implementation. They concluded that the cost of adopting robotics may be mitigated by a shorter learning curve compared with laparoscopy. Consistently with this, they found no significant cost differences between the robotic group and the mastery phase group, while overall total costs for robotic liver surgery were even lower, mainly because of shorter hospital stay and fewer severe complications.

### Cost differences globally

Median costs were calculated for studies from Asia, the USA, and Europe. Median total costs were highest in studies from the USA, followed by Asia and Europe (Table 4). Regional cost estimates showed substantial variation across studies. However, these differences were interpreted descriptively only, as cross-regional comparisons were limited by heterogeneity in healthcare systems, cost-accounting methods, reimbursement structures, and included cost components.

**Table 4:** Cost medians of robotic liver resection (RLR) between Asian, American and European countries

	Asia	USA	Europe
Median RLR total costs	18,260	39,250	18,970
Median RLR intraoperative costs	15,030	10,820	4850
Median RLR hospitalization costs	2120	12,950	7520

### Readmission and follow-up costs

A minority of studies reported readmission-related costs or readmission rates (*n* = 7, 21.9%). Comparability was limited because the time horizon for readmission analyses varied substantially, ranging from 30 days to 6 months. Only one study assessed longer-term costs, such as outpatient expenses. Overall, six of the seven studies found no significant differences in readmission between the three surgical approaches.

### Quality assessment

**Fig. 6:**
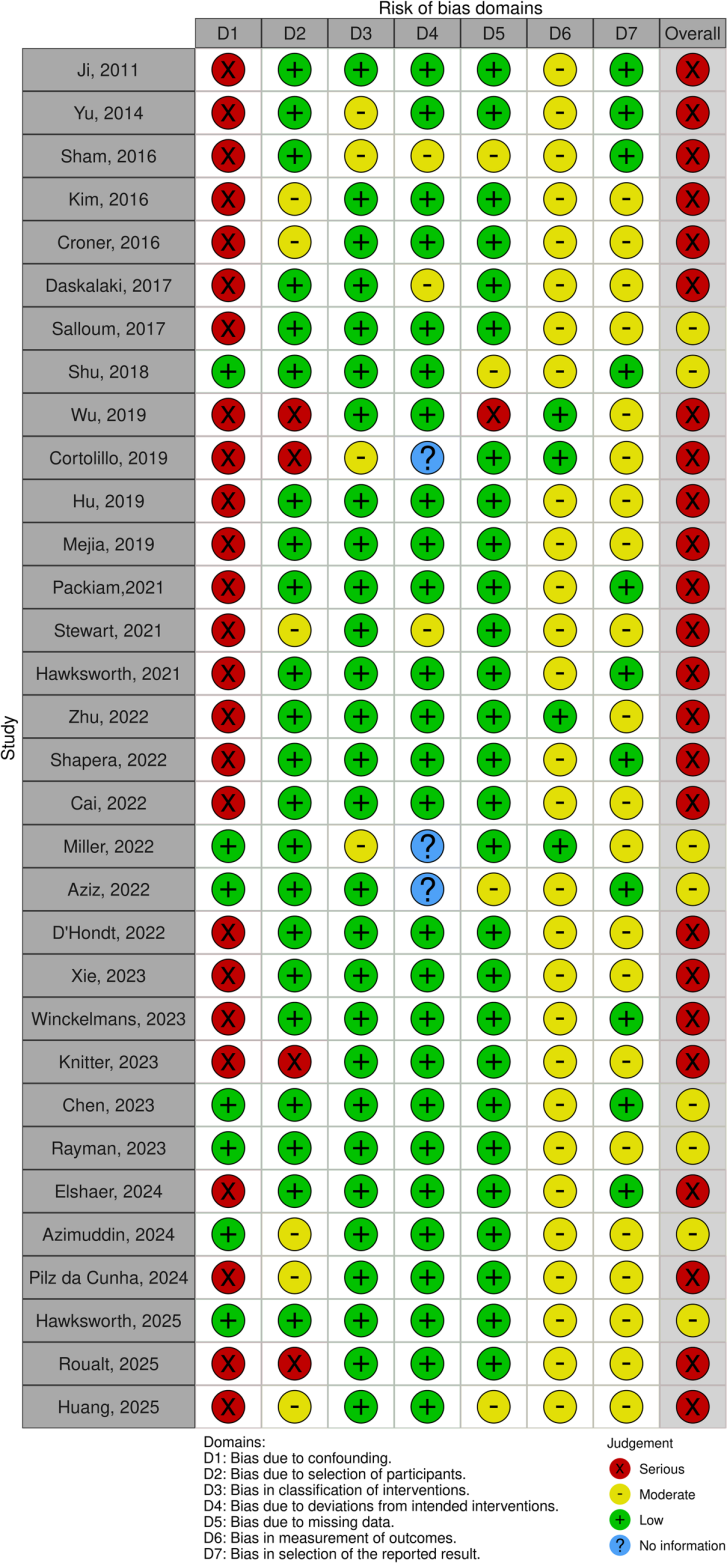
ROBINS-I risk of bias assessments

All 32 studies underwent quality assessment using ROBINS-I and the Drummond Checklist, with the full results shown graphically in Table 5 and Fig. 6.

**Table 5:** Heat map of quality assessment results using the Drummond checklist

Study	Research question	Description of alternatives	Effectiveness of program	Important costs & consequences	Costs & consequence measures	Costs & consequence values	Differential timing adjustment	Incremental analysis	Allowance for uncertainty	Presentation & discussion	Score
Huang et al.	1	1	1	0	1	1	0	0	0	1	6
Roualt et al.	1	1	1	1	1	1	0	0	0	1	7
Hawksworth et al.	1	1	1	0	1	1	0	0	0	1	6
Pilz da Cunha et al.	1	1	1	1	1	1	1	0	1	1	9
Azimuddin et al.	1	1	1	1	1	1	1	0	0	1	8
Elshaer et al.	1	1	1	0	1	1	0	0	0	0	5
Rayman et al.	1	1	1	0	1	1	0	0	0	0	5
Chen et al.	1	1	1	0	0	0	0	0	0	0	3
Knitter et al.	1	1	1	0	1	1	0	0	0	1	6
Winckelmans et al.	1	1	1	0	1	1	0	0	0	1	6
Xie et al.	1	1	1	0	0	0	0	0	0	1	4
Cai et al.	1	1	1	0	0	0	0	0	0	1	4
Shapera et al.	1	1	1	0	1	1	0	0	0	1	6
Zhu et al.	1	1	1	0	0	0	0	0	0	1	4
D´Hondt	1	1	1	0	1	1	0	0	0	1	6
Aziz et al.	1	1	1	0	0	1	0	0	0	0	4
Miller et al.	1	1	1	0	1	1	0	1	0	1	7
Hawksworth et al.	1	1	1	0	1	1	0	0	0	1	6
Stewart et al.	1	1	1	0	1	1	0	0	0	1	6
Peckiam et al.	1	1	1	1	1	1	0	0	0	1	7
Mejia et al.	1	1	1	0	1	1	0	0	0	1	6
Hu et al.	1	1	1	0	0	0	0	0	0	1	4
Wu et al.	1	1	1	0	1	1	0	0	0	1	6
Cortolillo et al.	1	1	1	0	0	1	0	0	0	1	5
Shu et al.	1	1	1	0	0	1	0	0	0	1	5
Salloum et al.	1	1	1	1	1	1	0	0	0	1	7
Daskalaki et al.	1	1	1	1	1	1	0	0	0	1	7
Croner et al.	1	1	1	1	1	1	0	0	0	1	7
Kim et al.	1	1	1	0	0	1	0	0	0	1	5
Sham et al.	1	1	1	1	1	1	0	0	0	1	7
Yu et al	1	1	1	0	0	1	0	0	0	1	5
Ji et al.	1	1	1	0	0	1	0	0	0	1	5

ROBINS-I assessment showed that most included studies were at moderate or serious risk of bias. 8 studies (*n* = 8/32, 25%) were deemed at moderate risk and 24 studies (*n* = 24/32, 75%) were deemed at serious risk of bias. The most common factor in serious risk of bias assessment was the presence of confounders, as treatment allocation was generally non-randomized and often influenced by case complexity, surgeon preference, and institutional or temporal factors. Studies using propensity score matching were judged as moderate risk of bias, whereas unadjusted or univariate adjusted retrospective comparisons and historical cohort studies were rated as serious risk of bias. Overall, bias in intervention classification, missing data, and outcome measurement was usually low, while selection bias and selective reporting were additional moderate risk concerns.

For the Drummond Checklist assessment, the average satisfied criteria were 5.8 out of 10 (range 3–9). The least satisfied criteria included adjustment for differential timing, the use of incremental analysis, and assessment of uncertainty. There were no studies which utilized quality-adjusted life years (QALYs) to calculate an Incremental Cost-Effectiveness Ratio (ICER). No study performed a formal cost-utility or cost-effectiveness analysis. 8 studies (*n* = 8/32, 25%) included indirect costs, such as initial robotic platform purchase, to satisfy the important and relevant costs criteria.

## Discussion

### Main findings

The current literature includes a range of economic evaluations for RLR with heterogeneous methods for cost reporting. When reported, intraoperative costs were generally higher for RLR than for LLR or OLR. However, studies assessing total costs and accounting for factors such as LOS, complication rates, and readmissions often found RLR to be cost-saving compared with OLR. LOS was consistently shorter after RLR than after OLR (and confirmed by meta-analysis), whereas this advantage was not consistently observed when RLR was compared with LLR. Total costs of RLR were shown to be higher than those of LLR through a meta-analysis. Although below our pre-specified threshold, heterogeneity remained substantial (I^2^ = 72%), and pooled cost estimates should be considered hypothesis-generating rather than definitive. Subgroup analyses of complex cases, such as major hepatectomies, showed no significant cost differences between the two approaches. The cost difference between RLR and LLR also decreased following learning phases in centers adopting the robotic approach. Indirect costs related to robotic platform purchase and maintenance were considered in only a minority of studies (25%). In particular, several studies did not capture important cost categories such as consumables, readmission-related costs, or indirect costs associated with robotic platform acquisition and maintenance. Overall, the methodological quality of the available evidence was limited. ROBINS-I assessment indicated a moderate to serious risk of bias across all studies, mainly due to their retrospective, unblinded design and the substantial risk of confounders caused by patient selection for robotic surgery. According to our health economic checklist, no study reported quality-adjusted life years (QALYs) or incremental cost-effectiveness ratios (ICERs), which represents a major limitation in evaluating the true cost-effectiveness of RLR. Likewise, no study performed a formal cost-utility or cost-effectiveness analysis. Therefore, this review evaluates economic burden and resource utilization rather than formal cost-effectiveness.

### Implications to current knowledge base

The findings of this systematic review were replicated by Koh et al. in their meta-analysis, featuring 45 studies of minimally invasive liver resections. The authors found that indirect costs were rarely reported in the literature. Their meta-analysis, including 20 studies which featured RLR groups, showed that LLR had the lowest total cost [49]. To further evaluate the economic value of minimally invasive liver surgery, they performed a cost–outcome analysis and demonstrated significant cost, morbidity, and efficacy advantages, driven primarily by shorter length of stay and lower complication rates. Since this meta-analysis, we include six additional studies to Koh et al. [19, 20, 22, 43, 46, 50], published in the last 2 years, which act as a potential signal that the cost of RLR is reducing in the modern era. Our analysis aimed to focus on novel and clinically relevant factors that are essential for assessing the cost impact of RLR. In particular, the subgroup analysis of complex cases is highly relevant, as these procedures have traditionally been considered a domain of open surgery and are often difficult or not feasible in the laparoscopic setting [43]. Our review considered center-volume-dependent cost patterns across multiple cost domains, with particular attention to complex cases, length of stay, learning curve, and case-volume effects. Robotic surgery may be particularly relevant in selected complex liver resections, where improved ergonomics and instrument dexterity could facilitate a minimally invasive approach. However, whether these potential technical and clinical advantages translate into consistent economic benefit remains uncertain. In our analysis, cost differences appeared to attenuate with increasing operative complexity and in studies accounting for learning curve or case-volume effects. Nevertheless, these observations are based on a limited number of heterogeneous retrospective studies and could not be formally pooled. Therefore, they should be interpreted cautiously as exploratory findings rather than definitive evidence of cost advantage.

The initial purchase and setup costs of robotic platforms are essential to complete a full economic analysis in the context of RLR. As more hepato-pancreato-biliary centers worldwide adopt the robotic approach, this cost information will better inform healthcare systems on the associated indirect costs, when planning surgical robotic programs. Complex liver resections are typically concentrated in high-volume centers with strong interdisciplinary infrastructure. In such settings, costs may be diluted more effectively. Although we found that indirect costs related to robotic surgery can be substantial, these may be offset in high-volume centers through greater case load, shared use of the robotic platform across departments, and experienced teams capable of reducing docking time and minimizing unnecessary use of costly disposables. Another important factor affecting costs is the learning curve. During the implementation phase, longer operative times and technical inefficiencies may increase procedural costs [19]. However, only a few studies accounted for learning curve effects, representing a potential confounding factor. Given that many included studies were conducted during relatively early phases of robotic adoption, this limitation is not unexpected. Nevertheless, studies from centers with extensive laparoscopic experience suggest that robotic surgery may have a shorter learning curve, likely because of its intuitive controls [20]. Emerging evidence suggests that RLR may be associated with lower conversion rates than LLR in complex cases, which may partially offset higher intraoperative costs and warrants more consistent inclusion in future economic evaluations [51].

### Strengths and limitations

The main challenge of addressing this research topic lies with the study heterogeneity. This was anticipated by our systematic review, and we have addressed this using a pragmatic approach. By performing exploratory meta-analysis, we have been able to provide pooled effect estimates where heterogeneity was deemed acceptable (I^2^ < 75%), in the comparisons of LOS and total costs of RLR and LLR. As anticipated, remaining comparisons were precluded by the high levels of heterogeneity. Our narrative synthesis of the results provides a solution to exploring the current cost differences within heterogeneous literature. It has allowed us to unearth the reported findings and provide important research recommendations.

The limitations of this review should be acknowledged. First, most included studies did not adequately account for the impact of case mix and procedural complexity on economic comparisons. This is particularly relevant in liver surgery, where differences in operative difficulty may substantially influence both intraoperative and postoperative costs. When data were available, our review analyzed the cost differences associated with case complexity and volume, showing that cost differences between RLR and other approaches reduced with increasing operative complexity. Second, the majority of studies adopted only short-term time horizons, thereby limiting the assessment of downstream economic consequences such as readmissions, outpatient care, and other longer-term costs. Time horizon is crucial in cost analyses, as an approach that appears cost-saving during the initial hospitalization may become less favorable if postoperative morbidity leads to frequent readmissions. Most studies did not adequately account for readmissions, which is a major weakness in evaluating true costs [21, 23]. Most studies reported a shorter length of stay for minimally invasive surgery compared with open surgery, which appeared to be a major driver of cost reduction. However, this decrease in length of stay may also be influenced by factors such as fast-track or enhanced recovery pathways and may therefore overestimate the direct effect of the operative approach on costs.

All included studies were retrospective in design. Three were based on national databases, which mainly capture charges rather than true costs. Although these large database studies tended to support the use of robotic surgery compared with laparoscopy or open surgery, they lacked granular cost accounting and may have been subject to misclassification of case complexity. Their findings should therefore be interpreted with caution. The remaining studies were single-center analyses and consequently carry a substantial risk of selection bias and limited generalizability.

Finally, there was considerable heterogeneity in cost terminology across studies. Some reported charges, others reported fees, reimbursements, or estimated costs. Given that many studies originated from the United States, where the reimbursement and costing framework differs fundamentally from those in Europe and Asia, cross-study and cross-regional comparability remains limited.

## Conclusion

In conclusion, laparoscopy remains the lowest-cost surgical approach for liver resection in the majority of the literature. The cost differences associated with the robotic approach appear to be volume- and center-maturity-dependent. While low-volume centers may face high fixed costs and inefficient implementation, high-volume, experienced centers may achieve economic viability through cost dilution, shorter hospital stay, and lower conversion rates, particularly in complex cases. This center-volume-dependent framework may provide a useful conceptual model for future economic evaluations of robotic liver surgery.

## Appendix

### Systematic review search strategy

See Table 6.

**Table 6:** Systematic review search strategy for OVID

Search number	Keyword search strategy
1	Robotic surgery.mp
2	Robot assisted surgery.mp
3	Surgical robotics.mp
4	Robot assisted minimally invasive surgery.mp
5	Robotic assisted procedures.mp
6	1 or 2 or 3 or 4 or 5
7	Cost-effectiveness.mp
8	Analyses, cost–benefit.mp
9	Analysis, cost–benefit.mp
10	Analyses, cost benefit.mp
11	Cost benefit analysis.mp
12	Cost benefit analyses.mp
13	Effectiveness, cost.mp
14	Cost–benefit data.mp
15	Cost utility analysis.mp
16	Cost utility analyses.mp
17	Analysis, cost-utility.mp
18	Analyses, cost-utility.mp
19	Economic evaluation.mp
20	Economic evaluations.mp
21	Evaluation, economic.mp
22	Evaluations, economic.mp
23	Cost effectiveness analysis.mp
24	Cost effectiveness analyses.mp
25	Analysis, cost-effectiveness.mp
26	Analyses, cost-effectiveness.mp
27	Cost.mp
28	Costs.mp
29	Cost effective.mp
30	Profit.mp
31	Profitability.mp
32	Cost-efficiency.mp
33	Cost efficiency.mp
34	Cost-efficient.mp
35	7 or 8 or 9 or 10 or 11 or 12 or 13 or 14 or 15 or 16 or 17 or 18 or 19 or 20 or 21 or 22 or 23 or 24 or 25 or 26 or 27 or 28 or 29 or 30 or 31 or 32 or 33 or 34
36	Hepatectom*.mp
37	Liver resection.mp
38	Hepatic resection.mp
39	36 or 37 or 38
40	6 and 35 and 39

### Cochrane library search strategy

(Robotic surgery or robot assisted surgery or surgical robotics or robot assisted minimally invasive surgery or robotic assisted procedures)

AND

(Cost-Effectiveness or Cost Effectiveness or Cost-Effectiveness Analysis or Cost-Effectiveness Analyses or Analysis, Cost-Effectiveness or Analyses, Cost-Effectiveness or Cost Benefit or Cost–Benefit Analysis or Cost–Benefit Analyses or Analysis, Cost–Benefit or Analyses, Cost–Benefit or Cost-Utility Analysis or Cost-Utility Analyses or Analysis, Cost-Utility or Analyses, Cost-Utility or Economic Evaluation or Economic Evaluations or Evaluation, Economic or Evaluations, Economic or Cost or Costs or Cost Effective or Cost Efficiency or Cost-efficiency or Cost-efficient or Profit or Profitability)

AND

(hepatectom* or liver resection or hepatic resection).

## References

[1] Wang K, Liu W, Yan XL, Li J, Xing BC (2017) Long-term postoperative survival prediction in patients with colorectal liver metastasis. Oncotarget 8(45):79927–79934 29108374 10.18632/oncotarget.20322 PMC5668107

[2] Petrowsky H, Fritsch R, Guckenberger M, De Oliveira ML, Dutkowski P, Clavien PA (2020) Modern therapeutic approaches for the treatment of malignant liver tumours. Nat Rev Gastroenterol Hepatol 17(12):755–772 32681074 10.1038/s41575-020-0314-8

[3] Clavien PA, Petrowsky H, DeOliveira ML, Graf R (2007) Strategies for safer liver surgery and partial liver transplantation. N Engl J Med 356(15):1545–1559 17429086 10.1056/NEJMra065156

[4] Mirnezami R, Mirnezami AH, Chandrakumaran K, Abu Hilal M, Pearce NW, Primrose JN, Sutcliffe RP (2011) Short- and long-term outcomes after laparoscopic and open hepatic resection: systematic review and meta-analysis. HPB (Oxford) 13(5):295–308 21492329 10.1111/j.1477-2574.2011.00295.x PMC3093641

[5] Machairas N, Papaconstantinou D, Tsilimigras DI, Moris D, Prodromidou A, Paspala A et al (2019) Comparison between robotic and open liver resection: a systematic review and meta-analysis of short-term outcomes. Updates Surg 71(1):39–48 30719624 10.1007/s13304-019-00629-0

[6] Ciria R, Berardi G, Alconchel F, Briceño J, Choi GH, Wu YM et al (2022) The impact of robotics in liver surgery: a worldwide systematic review and short-term outcomes meta-analysis on 2,728 cases. J Hepatobiliary Pancreat Sci 29(2):181–197 33200536 10.1002/jhbp.869

[7] Bray F, Laversanne M, Sung H, Ferlay J, Siegel RL, Soerjomataram I, Jemal A (2022) Global cancer statistics 2022: GLOBOCAN estimates of incidence and mortality worldwide for 36 cancers in 185 countries. CA Cancer J Clin 74(3):229–263 10.3322/caac.21834 38572751

[8] Abu Hilal M, Hoogteijling TJ, Edwin B, Dagher I, D’Hondt M, Marques HP et al (2025) The Brescia internationally validated European guidelines on minimally invasive liver surgery. Br J Surg 112(6):znaf113 40568914 10.1093/bjs/znaf113 PMC12199258

[9] Shea BJ, Reeves BC, Wells G, Thuku M, Hamel C, Moran J et al (2017) AMSTAR 2: a critical appraisal tool for systematic reviews that include randomised or non-randomised studies of healthcare interventions, or both. BMJ 358:j4008 28935701 10.1136/bmj.j4008 PMC5833365

[10] Page MJ, McKenzie JE, Bossuyt PM, Boutron I, Hoffmann TC, Mulrow CD et al (2021) The PRISMA 2020 statement: an updated guideline for reporting systematic reviews. BMJ 372:n71 33782057 10.1136/bmj.n71 PMC8005924

[11] Campbell M, Katikireddi SV, Sowden A, McKenzie JE, Thomson H (2018) Improving conduct and reporting of narrative synthesis of quantitative data (ICONS-Quant): protocol for a mixed methods study to develop a reporting guideline. BMJ Open 8(2):e020064

[12] Wan X, Wang W, Liu J, Tong T (2014) Estimating the sample mean and standard deviation from the sample size, median, range and/or interquartile range. BMC Med Res Methodol 14:135 25524443 10.1186/1471-2288-14-135 PMC4383202

[13] Hozo SP, Djulbegovic B, Hozo I (2005) Estimating the mean and variance from the median, range, and the size of a sample. BMC Med Res Methodol 5(1):13 15840177 10.1186/1471-2288-5-13 PMC1097734

[14] Higgins JP, Altman DG, Gøtzsche PC, Jüni P, Moher D, Oxman AD et al (2011) The Cochrane collaboration’s tool for assessing risk of bias in randomised trials. BMJ 343:d5928 22008217 10.1136/bmj.d5928 PMC3196245

[15] McGuinness LA, Higgins JPT (2021) Risk-of-bias VISualization (robvis): an R package and shiny web app for visualizing risk-of-bias assessments. Res Synth Methods 12(1):55–61 32336025 10.1002/jrsm.1411

[16] Drummond MF, Jefferson TO (1996) Guidelines for authors and peer reviewers of economic submissions to the BMJ. The BMJ economic evaluation working party. BMJ 313(7052):275–83 8704542 10.1136/bmj.313.7052.275 PMC2351717

[17] Stewart C, Wong P, Warner S, Raoof M, Singh G, Fong Y, Melstrom L (2021) Robotic minor hepatectomy: optimizing outcomes and cost of care. HPB 23(5):700–706 32988754 10.1016/j.hpb.2020.09.005

[18] Sham JG, Richards MK, Seo YD, Pillarisetty VG, Yeung RS, Park JO (2016) Efficacy and cost of robotic hepatectomy: is the robot cost-prohibitive? J Robot Surg 10(4):307–313 27153838 10.1007/s11701-016-0598-4

[19] Rouault A, Pecquenard F, Elamrani M, Boleslawski E, Truant S, Millet G (2025) Cost-effectiveness and clinical impact of robotic-assisted hepatectomy. J Robot Surg 19(1):156 40229619 10.1007/s11701-025-02319-z PMC11997018

[20] Hawksworth J, Radkani P, Shoucair S, Gogna S, Fishbein T, Winslow E (2025) One hundred and fifty-two robotic hepatectomies at a North American hepatobiliary program: evolution of practice, learning curve, appraisal of outcomes, and cost analysis. Surg Endosc 39(3):2136–2146 39966129 10.1007/s00464-025-11570-2

[21] Cortolillo N, Patel C, Parreco J, Kaza S, Castillo A (2019) Nationwide outcomes and costs of laparoscopic and robotic vs. open hepatectomy. J Robot Surg 13(4):557–565 30484059 10.1007/s11701-018-0896-0

[22] Azimuddin AM, Hirata Y, Boyev A, Jain AJ, Ayabe R, Ajith J et al (2024) A propensity score matched cost analysis of robotic versus open hepatectomy. HPB (Oxford) 26(11):1379–1386 39198140 10.1016/j.hpb.2024.08.001

[23] Aziz H, Hanna K, Lashkari N, Ahmad N-U-S, Genyk Y, Sheikh MR (2022) Hospitalization costs and outcomes of open, laparoscopic, and robotic liver resections. Am Surg 88(9):2331–2337 33861658 10.1177/00031348211011063

[24] Xie F, Wang D, Ge J, Liao W, Li E, Wu L, Lei J (2023) Robotic approach together with an enhanced recovery programme improve the perioperative outcomes for complex hepatectomy. Front Surg 10:1135505 37334205 10.3389/fsurg.2023.1135505 PMC10272522

[25] Wu C-Y, Chen P-D, Chou W-H, Liang J-T, Huang C-S, Wu Y-M (2019) Is robotic hepatectomy cost-effective? In view of patient-reported outcomes. Asian J Surg 42(4):543–550 30704965 10.1016/j.asjsur.2018.12.010

[26] Shu J, Wang XJ, Li JW, Bie P, Chen J, Zheng SG (2019) Robotic-assisted laparoscopic surgery for complex hepatolithiasis: a propensity score matching analysis. Surg Endosc 33(8):2539–2547 30350102 10.1007/s00464-018-6547-8

[27] Ji WB, Wang HG, Zhao ZM, Duan WD, Lu F, Dong JH (2011) Robotic-assisted laparoscopic anatomic hepatectomy in China: initial experience. Ann Surg 253(2):342 21135692 10.1097/SLA.0b013e3181ff4601

[28] Croner RS, Perrakis A, Hohenberger W, Brunner M (2016) Robotic liver surgery for minor hepatic resections: a comparison with laparoscopic and open standard procedures. Langenbecks Arch Surg 401(5):707–714 27207697 10.1007/s00423-016-1440-1

[29] Ziogas IA, Evangeliou AP, Mylonas KS, Athanasiadis DI, Cherouveim P, Geller DA et al (2021) Economic analysis of open versus laparoscopic versus robotic hepatectomy: a systematic review and meta-analysis. Eur J Health Econ 22(4):585–604 33740153 10.1007/s10198-021-01277-1

[30] Knitter S, Feldbrügge L, Nevermann N, Globke B, Galindo SAO, Winklmann T et al (2023) Robotic versus laparoscopic versus open major hepatectomy – an analysis of costs and postoperative outcomes in a single-center setting. Langenbecks Arch Surg 408(1):214 37247050 10.1007/s00423-023-02953-x PMC10226911

[31] Hawksworth J, Llore N, Holzner ML, Radkani P, Meslar E, Winslow E et al (2021) Robotic hepatectomy is a safe and cost-effective alternative to conventional open hepatectomy: a single-center preliminary experience. J Gastrointest Surg 25(3):825–828 33001352 10.1007/s11605-020-04793-2

[32] Daskalaki D, Gonzalez-Heredia R, Brown M, Bianco FM, Tzvetanov I, Davis M et al (2017) Financial impact of the robotic approach in liver surgery: a comparative study of clinical outcomes and costs between the robotic and open technique in a single institution. J Laparoendosc Adv Surg Tech A 27(4):375–382 28186429 10.1089/lap.2016.0576 PMC5397272

[33] Shapera E, Sucandy I, Syblis C, Crespo K, Ja’Karri T, Ross S, Rosemurgy A (2022) Cost analysis of robotic versus open hepatectomy: is the robotic platform more expensive? J Robot Surg 16(6):1409–1417 35152343 10.1007/s11701-022-01375-z

[34] Mejia A, Cheng SS, Vivian E, Shah J, Oduor H, Archarya P (2020) Minimally invasive liver resection in the era of robotics: analysis of 214 cases. Surg Endosc 34(1):339–348 30937618 10.1007/s00464-019-06773-3

[35] Zhu L, Liu Y, Hu M, Zhao Z, Li C, Zhang X et al (2022) Comparison of robotic and laparoscopic liver resection in ordinary cases of left lateral sectionectomy. Surg Endosc 36(7):4923–4931 34750706 10.1007/s00464-021-08846-8

[36] Yu Y-D, Kim K-H, Jung D-H, Namkoong J-M, Yoon S-Y, Jung S-W et al (2014) Robotic versus laparoscopic liver resection: a comparative study from a single center. Langenbecks Arch Surg 399(8):1039–1045 25366357 10.1007/s00423-014-1238-y

[37] Miller HP, Hakim A, Kellish A, Wozniak M, Gaughan J, Sensenig R et al (2022) Cost-benefit analysis of robotic vs. laparoscopic hepatectomy: a propensity-matched retrospective cohort study of American college of surgeons national surgical quality improvement program database. Am Surg 88(12):2886–2892 33861656 10.1177/00031348211011124

[38] Kim JK, Park JS, Han DH, Choi GH, Kim KS, Choi JS, Yoon DS (2016) Robotic versus laparoscopic left lateral sectionectomy of liver. Surg Endosc 30(11):4756–4764 26902613 10.1007/s00464-016-4803-3

[39] Hu M, Liu Y, Li C, Wang G, Yin Z, Lau WY, Liu R (2019) Robotic versus laparoscopic liver resection in complex cases of left lateral sectionectomy. Int J Surg 67:54–60 31121328 10.1016/j.ijsu.2019.05.008

[40] Cai J-P, Chen W, Chen L-H, Wan X-Y, Lai J-M, Yin X-Y (2022) Comparison between robotic-assisted and laparoscopic left hemi-hepatectomy. Asian J Surg 45(1):265–268 34120821 10.1016/j.asjsur.2021.05.017

[41] Winckelmans T, Wicherts DA, Parmentier I, De Meyere C, Verslype C, D’Hondt M (2023) Robotic versus laparoscopic hepatectomy: a single surgeon experience of 629 consecutive minimally invasive liver resections. World J Surg 47(9):2241–2249 37208537 10.1007/s00268-023-07060-y

[42] D’Hondt M, Devooght A, Willems E, Wicherts D, De Meyere C, Parmentier I et al (2023) Transition from laparoscopic to robotic liver surgery: clinical outcomes, learning curve effect, and cost-effectiveness. J Robot Surg 17(1):79–88 35322342 10.1007/s11701-022-01405-w

[43] Pilz da Cunha G, Coupé VMH, Zonderhuis BM, Bonjer HJ, Erdmann JI, Kazemier G et al (2024) Healthcare cost expenditure for robotic versus laparoscopic liver resection: a bottom-up economic evaluation. HPB (Oxford) 26(8):971–980 38853074 10.1016/j.hpb.2024.05.017

[44] Chen W, Zhang X, Jiang J, Ye Y, Zhai Z, Hu W et al (2023) Robotic versus laparoscopic liver resection in posterosuperior region: a retrospective study of consecutive cases. Surg Endosc 37(6):4728–4736 36890414 10.1007/s00464-023-09952-5

[45] Salloum C, Lim C, Lahat E, Gavara CGI, Levesque E, Compagnon P, Azoulay D (2017) Robotic-assisted versus laparoscopic left lateral sectionectomy: analysis of surgical outcomes and costs by a propensity score matched cohort study. World J Surg 41(2):516 27743071 10.1007/s00268-016-3736-2

[46] Huang C-T, Lin C-C, Lee K-T, Chang W-T (2025) The predictors of intraoperative surgical expenses in liver resection for hepatocellular carcinoma. Formos J Surg 58(1):18–24

[47] Rayman S, Sucandy I, Ross SB, Crespo K, Syblis C, Rosemurgy A (2023) A propensity score matched analysis of robotic and open hepatectomy for treatment of liver tumors. Clinical outcomes, oncological survival, and costs comparison. J Robot Surg 17(5):2399–2407 37428364 10.1007/s11701-023-01674-z

[48] Packiam V, Bartlett DL, Tohme S, Reddy S, Marsh JW, Geller DA, Tsung A (2012) Minimally invasive liver resection: robotic versus laparoscopic left lateral sectionectomy. J Gastrointest Surg 16(12):2233–2238 23054901 10.1007/s11605-012-2040-1 PMC3509231

[49] Koh YX, Zhao Y, Tan IE, Tan HL, Chua DW, Loh WL et al (2024) Comparative cost-effectiveness of open, laparoscopic, and robotic liver resection: a systematic review and network meta-analysis. Surgery 176(1):11–23 38782702 10.1016/j.surg.2024.04.015

[50] Elshaer M, Askari A, Pathanki A, Rajani J, Ahmad J (2024) Comparative study of operative expenses: robotic vs. laparoscopic vs. open liver resections at a university hospital in the UK. J Robot Surg 18(1):12 38214790 10.1007/s11701-023-01778-6

[51] Wang P, Zhang D, Huang B, Zhou WH, Wang CS, Zhao SY et al (2025) Robotic versus laparoscopic hepatectomy: meta-analysis of propensity-score matched studies. BJS Open 9(2):zrae141 40164991 10.1093/bjsopen/zrae141 PMC11957917

